# Blocking DNA Damage Repair May Be Involved in Stattic (STAT3 Inhibitor)-Induced FLT3-ITD AML Cell Apoptosis

**DOI:** 10.3389/fcell.2021.637064

**Published:** 2021-03-16

**Authors:** Yuxuan Luo, Ying Lu, Bing Long, Yansi Lin, Yanling Yang, Yichuang Xu, Xiangzhong Zhang, Jingwen Zhang

**Affiliations:** ^1^Department of Pediatric, Guangzhou Women and Children’s Medical Center, Guangzhou, China; ^2^Department of Hematology, Third Affiliated Hospital of Sun Yat-sen University, Guangzhou, China; ^3^Department of Blood Transfusion, Third Affiliated Hospital of Sun Yat-sen University, Guangzhou, China; ^4^Sen Yat-sen Institute of Hematology, Guangzhou, China; ^5^Department of General Medicine, Sun Yat-sen Memorial Hospital, Sun Yat-sen University, Guangzhou, China

**Keywords:** FLT3-ITD mutation, acute myeloid leukemia, DNA damage repair, apoptosis, STAT3

## Abstract

The FMS-like tyrosine kinase 3 (FLT3)- internal tandem duplication (ITD) mutation can be found in approximately 25% of all acute myeloid leukemia (AML) cases and is associated with a poor prognosis. The main treatment for FLT3-ITD-positive AML patients includes genotoxic therapy and FLT3 inhibitors, which are rarely curative. Inhibiting STAT3 activity can improve the sensitivity of solid tumor cells to radiotherapy and chemotherapy. This study aimed to explore whether Stattic (a STAT3 inhibitor) affects FLT3-ITD AML cells and the underlying mechanism. Stattic can inhibit the proliferation, promote apoptosis, arrest cell cycle at G0/G1, and suppress DNA damage repair in MV4-11cells. During the process, through mRNA sequencing, we found that DNA damage repair-related mRNA are also altered during the process. In summary, the mechanism by which Stattic induces apoptosis in MV4-11cells may involve blocking DNA damage repair machineries.

## Introduction

FMS-like tyrosine kinase 3 (FLT3) mutations include the FLT3-internal tandem duplication (ITD) mutation (approximately 25% of all AML cases) and FLT3-tyrosine kinase domain mutation (approximately 7–10% of all AML cases). FLT3-ITD is a common driver mutation that presents a high leukemic burden and is associated with a poor prognosis in patients with AML (Daver et al., 2019). The FLT3-ITD mutation is the insertion of a repetitive segment of the gene encoding the membrane region of the FLT3 receptor. Its length varies from 3 to >400 base pairs, and the number of inserted bases is usually a multiple of three (Levis and Small, 2003). The MV4-11 cell line is a FLT3-ITD mutant leukemia cell line which is often used in research related to FLT3-ITD AML (Quentmeier et al., 2003).

Genome instability is one of the mechanisms of drug resistance. Previous studies have found that in FLT3-ITD AML cells, there is high spontaneous DNA damage and both the micro-homology-mediated alternative non-homologous end-joining (Alt-NHEJ) and homologous recombination (HR) pathways are active (Fan et al., 2010). Alt-NHEJ is error-prone and leads to gene mutations. Although the HR pathway has high accuracy, it often leads to loss of heterozygosity (LOH). Clinically, patients with FLT3-ITD mutation recurrence often have new cytogenetic and molecular abnormalities and a higher FLT3-ITD/FLT3-wild-type (FLT3-WT) ratio (Stirewalt et al., 2014).

Radiotherapy and most chemotherapies induce apoptosis by causing DNA double-strand breaks (DSBs) in tumor cells. DNA damage repair (DDR) is initiated after the cell is genetically damaged. If DDR is not activated, the cell undergoes apoptosis (Takagi, 2017). Radiotherapy or chemotherapy resistance is related to the activity of DDR (Huang and Zhou, 2020). Inhibiting the repair of DNA damage in tumor cells is crucial for solving the drug resistance of tumor cells (Goldstein and Kastan, 2015).

Signal transducer and activator of transcription 3 (STAT3) is a vital regulatory factor of signal transduction and transcriptional activation and is essential for the proliferation, differentiation, and apoptosis of cells (Wang et al., 2018). In normal cells, the activation of STAT3 is rapid and transient; however, numerous studies have confirmed that the abnormal activation of STAT3 is involved in the development of tumors. In leukemia cells, abnormal expression and activation of STAT3 always occur, which accelerate proliferation, block differentiation, and inhibit apoptosis by inducing anti-apoptotic gene expression (Redell et al., 2011; Arora et al., 2018). A previous study found that the stimulation of FLT3-ITD AML cells (MV4-11) elevates p-STAT3 levels, which upregulates the expression of anti-apoptotic genes, thereby protecting AML cells from apoptosis (Zhou et al., 2009; Shi et al., 2018).

STAT3 is also related to the DDR process. Inhibiting its expression can increase the degree of DNA damage induced by radiation in tumor cells and promote apoptosis of tumor cells. Radiotherapy and chemotherapy promote apoptosis by causing DSBs (Takagi, 2017). For example, VP-16 (etoposide) is a traditional antitumor drug that acts on DNA topoisomerase II and promotes tumor cell apoptosis by inducing DSBs. However, many tumors are resistant to radiotherapy or chemotherapy because of active DDR pathways (Begg et al., 2011). STAT3 inhibitors can improve the sensitivity to radiotherapy and chemotherapy in solid tumors such as glioma (Han et al., 2016), esophageal cancer (Zhang et al., 2015), head and neck tumors (Adachi et al., 2012; Pan et al., 2013). Similarly, the decreased activity of STAT3 in mouse fibroblasts hindered the cell’s ability to repair DSBs induced by reactive oxygen species (ROS) (Barry et al., 2010). This suggests that STAT3 is a key factor in DDR.

Stattic is a selective STAT3 inhibitor, which inhibits the function of the STAT3 SH2 domain without affecting STAT1 (Schust et al., 2006). In this study, we used Stattic to inhibit the activity of STAT3, explore its effect on DDR in FLT3-ITD AML cells, and find possible ways to harness this effect.

## Materials and Methods

### Cell Lines and Reagents

Human AML cells HL60 and KG1a were obtained from the Guangzhou Institute of Biomedicine and Health, Chinese Academy of Sciences. Human AML cells MV4-11were obtained from the Shanghai Institutes of Biochemistry and Cell Biology, Chinese Academy of Sciences. All cell lines used in this examination were free of mycoplasma infection. Stattic was purchased from CAYMAN CHEMICAL COMPANY and dissolved in DMSO. VP16 was purchased from Sigma (United States) and diluted with DMSO.

### Antibodies

Annexin-V-PI antibody was purchased from BD (United States). The γ-H2AX antibody was purchased from BioLegend (United States). Western blotting-related antibodies GPADH, Phospho-Stat3 (Tyr705), Anti-rabbit IgG, HRP-linked Antibody were purchased from CST (United States).

### Flow Cytometry

Cells in the logarithmic growth phase were collected at a density of 1 × 10^6^ per well, washed twice with 2% FBS solution, blocked by Fc (Fc: 2% FBS = 1:100), and placed on ice for 10–15 min. Next, the antibodies (1:200) were added, and the cells were incubated for another 15 min in the dark. Moreover, the blank tube and single stain tubes were set. In the end, the fluorescence intensity was detected using BD Bioscience C6 flow cytometry and analyzed with Flowjo software.

### Cell Viability Assay

Cells were seeded at a density of 2.5 × 10^5^ to 5 × 10^5^ cells per mL and incubated in the presence or absence of Stattic for the indicated times. Cell viability was measured using the CCK8 assay kit according to the manufacturer’s instructions (DOJINDO, Japan). Cell line experiments were repeated three times. Data were analyzed using the GraphPad Prism software.

### DNA Sequencing

DNA was extracted using the Magen Hipure Tissue DNA Micro Kit (China) according to the manufacturer’s instructions. DNA sequencing was performed by Sangon Biotech according to their established protocol, using the following primer sequences: (5′–3′): Flt3: forward GCA ATT TAG GTA TGA AAG CCA GC reverse CTT TCA GCA TTT TGA CGG CAA CC. Data were analyzed using the SnapGene software.

### Western Blotting

Cells were lysed for 30 min in an ice-cold buffer containing RIPA lysate (approximately 5 × 10^5^ cells/ml), PMSF (1:100), leupetin (1:1,000), and NaVO_3_ (5:1,000), and the supernatant was collected. Subsequently, the OD value was obtained and the samples were prepared. Samples were electrophoresed at room temperature for 90 min and transferred on ice for 100 min; the required internal reference protein and target protein band were blocked at room temperature for 30 min to 1 h after being cut. Next, the samples were placed in TBST buffer with primary antibody overnight at 4°C followed by incubation with secondary antibody for 2 h and the addition of ECL solution. Finally, the samples were visualized using Image J software.

### qRT-PCR

RNA was extracted by using the TAKARA RNA extraction kit (Japan); purity and concentration were determined; reverse transcription reaction was performed using the TAKARA reverse transcription kit and the TAKARA kit for polymerase chain reaction. Finally, data analysis was performed using GAPDH as the baseline and the 2^–ΔΔ^ct value analysis. Primer sequences were as follows (5′–3′): STAT3: forward ACCAGCA GTATAGCCGCTTC reverse GCCACAATCCGGGCAATCTG APDH: forward ACCACAGTCCATGCCATCAC reverse TCC ACCACCCTGTTGCTGTA.

### RNA Sequencing Analysis

MV4-11 cells were treated with DMSO (control), Stattic (2.5 μM), or Stattic (2.5 μM) + VP16 (4 μg/ml) for 4.5 h. The experiment was repeated thrice. Transcriptome sequencing was conducted by OE Biotech Co., Ltd. (Shanghai, China) according to their established protocol. Briefly, total RNA was extracted from the sample; DNA was digested with DNase; and the eukaryotic mRNA was enriched with magnetic beads with Oligo (dT). Interruption reagents were added to obtain short fragments of mRNA. Using the interrupted mRNA as a template, a six-base random primer was used to synthesize the first-strand cDNA, and then the second-strand formation reaction system was prepared to synthesize the second-strand cDNA, and the double-stranded cDNA was purified using the Stranded cDNA kit. The purified double-stranded cDNA was subjected to end repair, a tail was added, the sequencing adapter was connected, the fragment size was selected, and finally, PCR amplification was performed. Agilent 2100 Bioanalyzer was used to control the quality of the constructed library. Illumina sequencer was then used for sequencing.

### Image Processing and Statistical Analysis

Images were processed using Adobe Illustrator and GraphPad Prism. All data are expressed as means ± *SD*. All data were analyzed using SPSS Statistics 16.0 or GraphPad Prism software. One-way ANOVA was used for comparisons among groups and LSD-test was used for comparisons between two groups. The values were considered significant at ^∗^*p* < 0.05, ^∗∗^*p* < 0.01, ^∗∗∗^*p* < 0.001, and ^****^p < 0.0001.

## Results

### Confirmation of FLT3 Gene Sequence in Cell Lines

**FIGURE 1:**
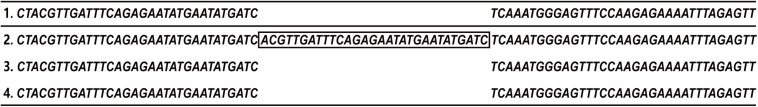
Confirmation of FLT3 gene sequence in cell lines. Partial FLT3 sequences of each cell line (1. FLT3-WT sequence from NCBI; 2. MV4-11 3. HL60 4. KG1a).

The MV4-11 cell line is often used in the study of FLT3-ITD mutant AML. First, DNA sequencing was used to confirm the sequences of the FLT3 gene in the MV4-11, KG1a, and HL60 cell lines. The results showed that the FLT3 sequences of KG1a and HL60 cell lines were consistent with those of the wild-type, whereas FLT3 of MV4-11 cells had an inserted 30 base pair sequence (Figure 1). This confirmed the presence of the FLT3-ITD mutation in the MV4-11 cell line and that of FLT3-WT in the KG1a and HL60 cell lines.

### STAT3 Expression in MV4-11 and FLT3-WT Cell Lines and Inhibition of STAT3-pi by Stattic

**FIGURE 2:**
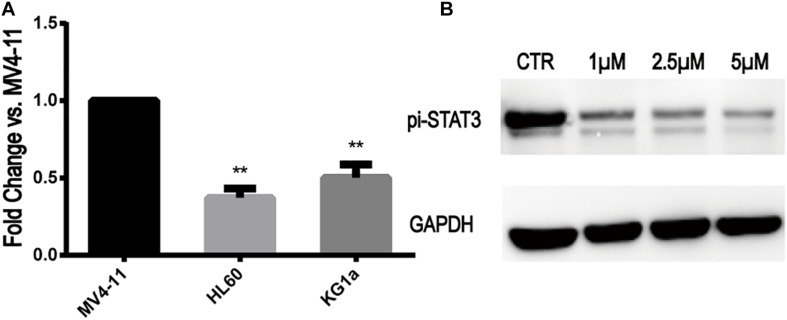
STAT3 expression in MV4-11 and FLT3-WT cell lines and inhibition of STAT3-pi by Stattic. **(A)** The expression of STAT3 mRNA in HL60 cell line was 0.37 ± 0.05-fold (*n* = 3) of MV4-11 cell line, and the expression of STAT3 mRNA in KG1a cell line was 0.50 ± 0.07-fold (*n* = 3) of MV4-11 cell line (**P < 0.01). **(B)** Protein expression of STAT3-pi in MV4-11 cell line treated with different concentrations of Stattic (0, 1, 2.5, and 5 μM), as detected by western blotting.

The expression of STAT3 mRNA levels in MV4-11 and FLT3-WT cell lines detected by qRT-PCR is shown in Figure 2A. The expression of STAT3 in MV4-11 cell line was higher than that in HL60 and KG1a cell lines.

Stattic is a selective STAT3 inhibitor, which inhibits the expression of STAT3-pi. MV4-11 cells were treated with DMSO (as control) or increasing concentrations of Stattic (1, 2.5, and 5 μM) for 24 h. Using western blotting, we determined that Stattic can effectively inhibit STAT3-pi (Figure 2B).

### Stattic Inhibits the Proliferation of MV4-11 Cells in a Dose- and Time-Dependent Manner

**FIGURE 3:**
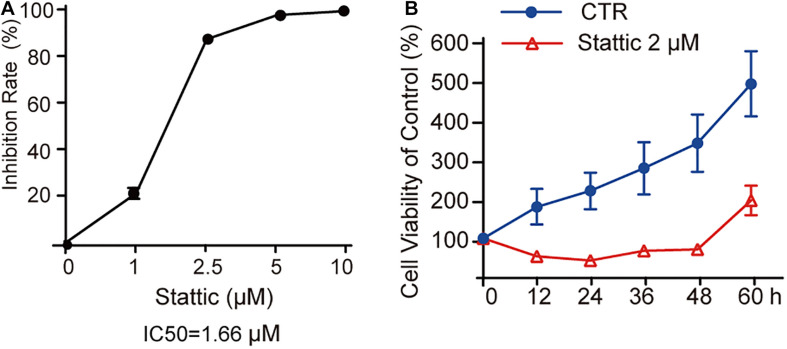
Stattic inhibits the proliferation of MV4-11 cells in a dose-and time-dependent manner. Stattic inhibits the growth of MV4-11 cells. The cell proliferation inhibition induced by Stattic in MV4-11 cells is time- and dose-dependent. **(A)** MV4-11 cells were treated with 0, 1, 2.5, 5, and 10 μM Stattic for 24 h (*n* = 3, IR of MV4-11 cells treated with 1, 2.5, 5, and 10 μM Stattic for 24 h were 21.00 ± 2.33%, 86.45 ± 0.58%, 96.76 ± 0.51%, and 98.54 ± 0.53%, respectively). **(B)** MV4-11 cells were treated with 2 μM Stattic or DMSO (as control) and cell viability was tested by CCK at 12, 24, 36, 48, and 60 h (*n* = 5, cell viability of DMSO group at 12, 24, 36, 48, and 60 h were 180.04 ± 44.39%, 219.92 ± 46.07%, 276.84 ± 65.26%, 340.23 ± 72.34%, and 489.21 ± 82.07%, respectively; cell viability of Stattic group at 12, 24, 36, 48, and 60 h were 54.85 ± 6.34%, 45.01 ± 3.49%, 68.66 ± 6.61%, 73.22 ± 4.76%, 196.21 ± 36.97%, respectively.

MV4-11 cells were treated with different concentrations of Stattic (1, 2.5, 5, and 10 μM) for 24 h and then subjected to a CCK8 assay to estimate the inhibition rate (IR) of cell proliferation. As shown in Figure 3A, the IR is dose-dependent, with an IC50 of 1.66 μM. When MV4-11 cells were treated with 2 μM Stattic or DMSO for 12, 24, 36, 48, and 60 h, the control group achieved higher proliferation than the Stattic group (Figure 3B), suggesting that the inhibition of cell proliferation by Stattic is dose- and time-dependent.

### Stattic Blocks the Cell Cycle in the G0/G1 Phase

**FIGURE 4:**
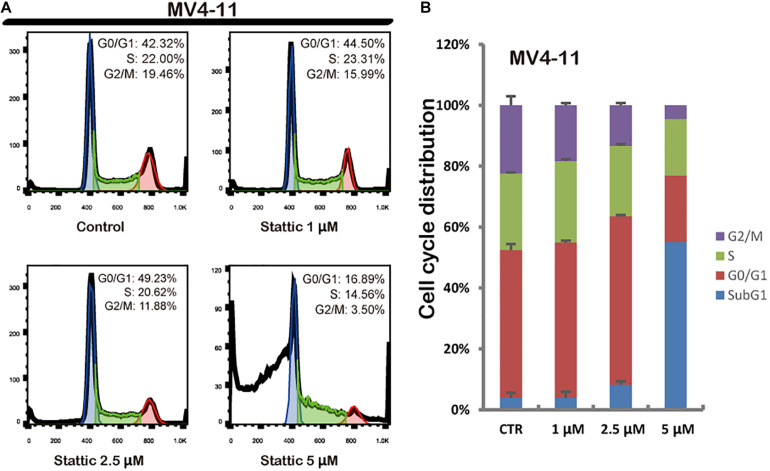
Stattic blocks the cell cycle in the G0/G1 phase. Cells were treated with 0, 1, 2.5, and 5 μM Stattic for 24 h. All cell cycle analyses were conducted thrice. The cell cycle distribution was determined using propidium iodide staining **(A,B)**.

Flow cytometry was performed to detect the cell cycle distribution of MV4-11 cells treated with DMSO (as control) or different concentrations of Stattic (1, 2.5, and 5 μM) for 24 h. The proportion of cells in the G0/G1 phase significantly increased, whereas the proportion of cells in the G2/M phase significantly decreased in the Stattic group compared with that in the control group, suggesting that Stattic blocks the cell cycle (Figure 4).

### Stattic Promotes Apoptosis in MV4-11 Cells

**FIGURE 5:**
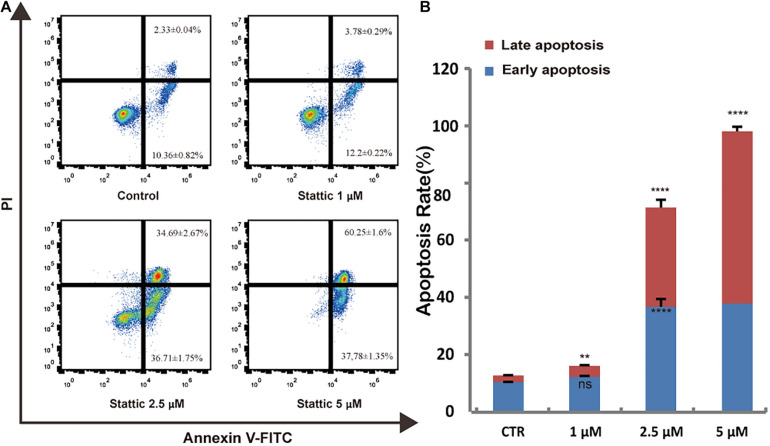
Stattic promotes apoptosis in MV4-11 cells. Cells were treated with 1, 2.5, and 5 μM Stattic for 24 h. Apoptosis were quantified by flow cytometry. Data from three replica plates were plotted. Data are shown as mean ± *SD* **(A,B)**. **p < 0.01 and ****p < 0.0001.

To further explore whether Stattic affects cell survival, we treated MV4-11 cells with DMSO (as control) or increasing concentrations of Stattic (1, 2.5, and 5 μM) for 24 h. Analysis of apoptosis using annexin-V/propidium iodide double staining showed that Stattic induces apoptosis in MV4-11 cells in a dose-dependent manner (Figure 5). Apoptosis rates are shown in Figure 5A.

### Stattic Induces DSBs in MV4-11 Cells

**FIGURE 6:**
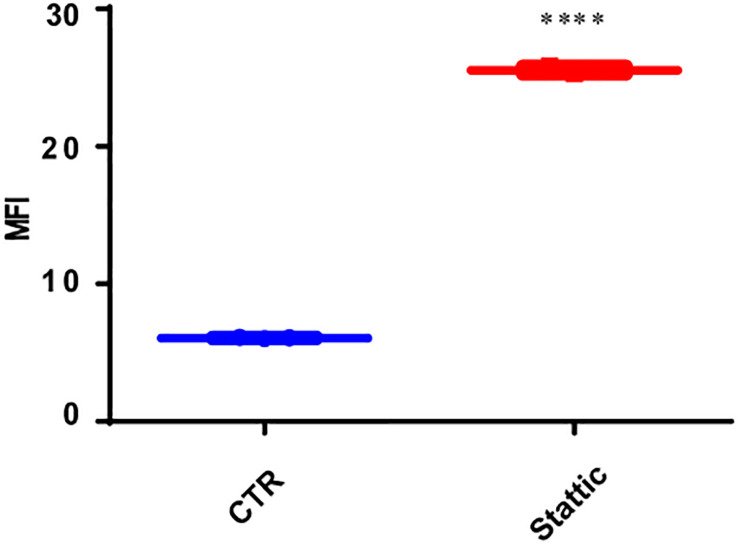
Stattic induces DSBs in MV4-11 cells. Cells were treated with Stattic (2.5 μM) or DMSO (as control) for 24 h (*n* = 3). The DSB level was determined using H2AX staining and measured by mean fluorescence intensity. ****p < 0.0001.

To explore the effect of Stattic on the induction of DSBs, we treated MV4-11 cells with Stattic (2.5 μM) or DMSO (control) for 24 h. The mean fluorescence intensity (MFI) of γ-H2AX detected by flow cytometry was used to evaluate the level of DSBs. As shown in Figure 6, the MFI of the Stattic-treated group was higher than that of the control group, suggesting that Stattic induces DSBs in MV4-11 cells.

### Stattic Combined With VP-16 Promote MV4-11 Apoptosis

**FIGURE 7:**
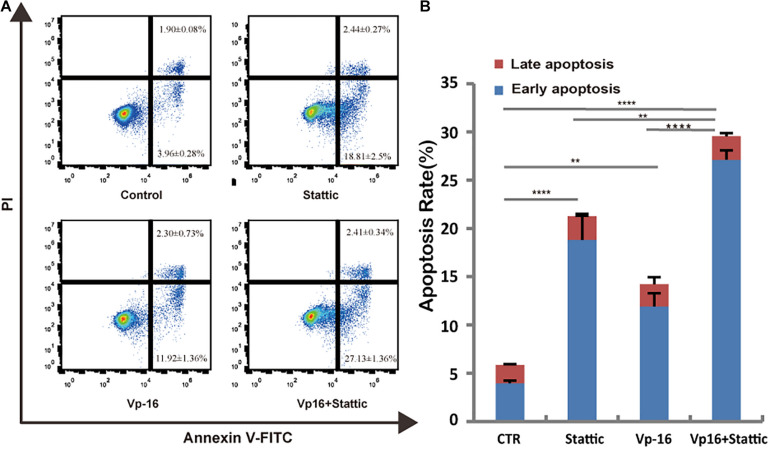
Stattic combined with VP-16 promote MV4-11 apoptosis. MV4-11 cells were treated with DMSO (control), Stattic (2.5 μM), and VP-16 (4 μg/ml) alone or in combination for 4.5 h. Apoptosis was measured by flow cytometry. Data from three replica plates were plotted. Apoptosis rates were shown as mean ± *SD* **(A,B)**. *p < 0.05, **p < 0.01, and ****p < 0.0001.

Vp-16, a traditional chemotherapy drug, is often used to treat leukemia. To explore the effect of Stattic combined with Vp-16 on FLT3-ITD mutant cells, we treated MV4-11 cells with Stattic (2.5 μM) and VP-16 (4 μg/ml) alone or in combination for 4.5 h. Apoptosis was then detected by flow cytometry. As shown in Figure 7, Stattic and VP-16 both promote apoptosis of MV4-11 cells. The early apoptosis, late apoptosis, and total apoptotic rates of the combined group were statistically increased compared with the VP16 or Stattic group.

### MV4-11 Cells Repaired DNA Damages

**FIGURE 8:**
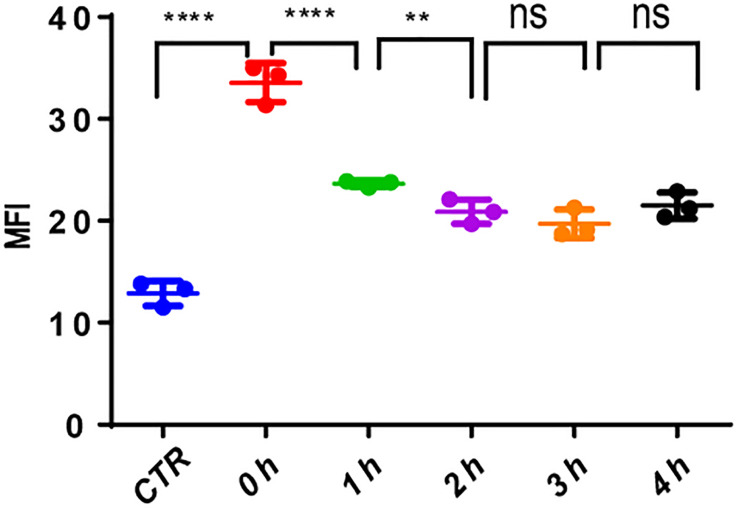
MV4-11 cells repaired DNA damages. MV4-11 cells were pretreated with VP-16 for 2 h to induce DSB and then washed off. Cells were then incubated in fresh culture medium for 0, 1, 2, 3, and 4 h. The MFI of γ-H2AX was detected by flow cytometry to evaluate the level of DSBs at each stage. MV4-11 cells not pretreated with VP-16 were used as control. **p < 0.01 and ****p < 0.0001.

The use of VP-16 causes DNA DSBs in leukemia cells; however, it is well-known that leukemia stem cells could repair DNA damage by DDR. To ascertain this, we pretreated MV4-11 cells with VP-16 and measured the MFI of γ-H2AX by flow cytometry over time. Right after drug elution, the MFI of pretreated cells was significantly higher than that of control cells, and it decreased gradually for 2 h. This indicates that MV4-11 cells can repair the DNA damage induced by VP-16 (Figure 8).

### Stattic Blocks DDR in MV4-11 Cells

**FIGURE 9:**
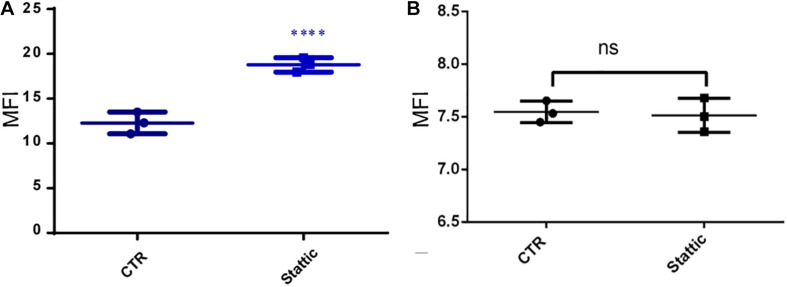
Stattic blocks DDR in MV4-11 cells. **(A)** MV4-11 cells pretreated with VP-16 for 2 h and then incubated in fresh culture medium with DMSO (as control) or Stattic (2.5 μM) for 4 h. The mean fluorescence intensity (MFI) of γ-H2AX was detected by flow cytometry to estimate DSBs levels in both groups (**A**, *n* = 3, ^****^P < 0.0001). **(B)** MV4-11 cells were treated with Stattic (2.5 μM) or DMSO (control) for 4 h. The MFI of γ-H2AX was detected to estimate DSB levels in both groups. There is no significant difference between the Stattic and control groups (**B**, *n* = 3. *P* > 0.5).

To investigate the effects of Stattic on the DNA repairing ability of MV4-11 cells, we induced DSBs using VP-16, and then cultured the cells in a medium containing Stattic and a medium without drug (control) for 4 h. We used flow cytometry to detect the expression of γH2AX, and the results are shown in Figure 9A: MFI of the Stattic group was maintained at a higher level than that of the control group. This indicated that Stattic hinders the repair of VP-16-induced DSBs by cells (Figure 9A). To further distinguish that the higher γH2AX expression in Stattic group is caused by delayed repair of VP16-induced damages but not by Stattic itself, MV4-11 cells were treated with Stattic or DMSO (control) for 4 h; the results are presented in Figure 9B. There is no significant difference between the Stattic and control groups, indicating that Stattic did not cause higher γH2AX at 4 h treatment (Figure 9B).

### Blocking DDR Participate in Apoptosis by Stattic in FLT3-ITD AML

**FIGURE 10:**
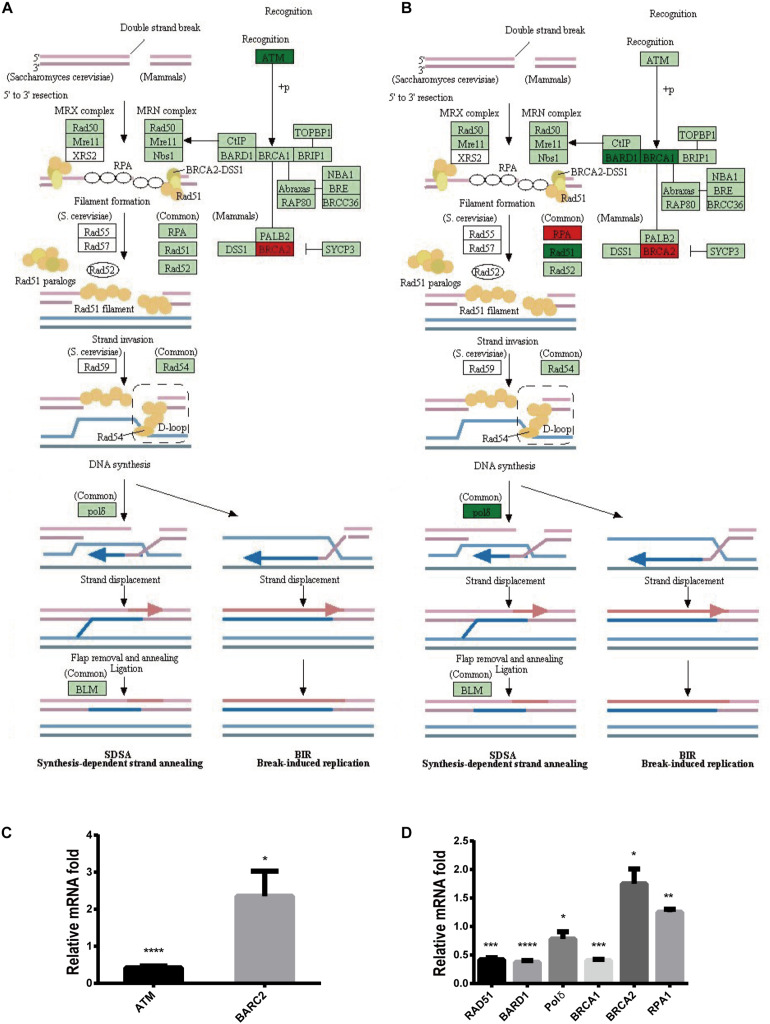
Blocking DDR is involved in apoptosis induced by Stattic in FLT3-ITD AML. MV4-11 cells were treated with DMSO (as control), Stattic (2.5 μM), or Stattic (2.5 μM) combined with VP-16 (4 μg/ml) for 4.5 h, and then mRNA sequencing was performed (*n* = 3). **(A)** Compared with the control group, the Stattic group has low ATM mRNA levels and BRCA2 mRNA is upregulated. **(B)** Compared with the control group, Stattic + VP-16 group had downregulated expression of BRCA1, RAD51, and polδ mRNA in the HR pathway (red: upregulated, green: downregulated, light green: unchanged). **(C)** The expression of ATMmRNA, BRCA2mRNA in Stattic group were 0.41 ± 0.04-fold, 2.35 ± 0.55-fold of control group. **(D)** The expression of BAD51mRNA, BARD1mRNA, polδmRNA, BRCA1mRNA, BRCA2mRNA, RPA1mRNA in Stattic + VP-16 group were 0.42 ± 0.03-fold, 0.37 ± 0.03-fold, 0.78 ± 0.10 -fold, 0.4 ± 0.02-fold, 1.75 ± 0.21-fold, 1.25 ± 0.04-fold of control group.RNA sequencing data can be obtained from the following URL: https://www.ncbi.nlm.nih.gov/sra/?term=PRJNA685978# repository accession number PRJNA685978. *p < 0.05, **p < 0.01, ***p < 0.001, and ****p < 0.0001.

To further explore the mechanism by which Stattic inhibits DDR in MV4-11 cells, MV4-11 cells were treated with different drugs [Stattic, Stattic combined with Vp-16, or DMSO (as control)] for 4.5 h, and then mRNA sequencing was performed. Data analysis was performed by OE Biotech Co., Ltd. (Shanghai, China). After KEGG analysis of genes with statistically significant changes in mRNA sequencing, it was found that in the DDR-related pathways (MMR, HR, etc. pathways), only the expression levels of some genes related to the HR pathway changed. The results showed that the Stattic group had lower ATM mRNA levels (Figures 10A,C). Cells treated with Stattic combined with VP-16 had a downregulated expression of BRCA1, RAD51, and polδ mRNA, which indicates that the combination of Stattic and VP-16 inhibited the expression of genes related to the HR pathway (Figures 10B,D). Therefore, the mechanism by which Stattic induces apoptosis in MV4-11 cells involves blocking the HR pathways of DDR pathways. However, the combined group shows upregulated expression of PRA and BRCA2, which are involved in the repair of DNA damage. The implications of this upregulation remain to be explored.

## Discussion

In 1995, Yu et al. (1995) discovered for the first time that the abnormal activation of STAT3 is related to the occurrence and development of tumors. This conclusion has been confirmed by other investigators. Activation of STAT3 is present in approximately 70% of all solid and hematological tumors (Kanna et al., 2018). STAT3 promotes the proliferation of leukemia cells through AKT/STAT3, Ras/Raf/MAPK and PI3K/AKT/mTOR, and other pathways (Badie et al., 1997). Simultaneously, aberrant STAT3 causes an increase in anti-apoptotic or a decrease in pro-apoptotic protein production and uncontrolled proliferation of cells (Kanna et al., 2018). Studies also found that inhibiting STAT3 activity can increase the degree of radiation damage to various solid tumor cells and promote tumor cell apoptosis (Pan et al., 2013; Zhang et al., 2015; Han et al., 2016).

Our experiments found that STAT3 inhibitors (Stattic) can effectively inhibit the proliferation of MV4-11 AML cells and promote apoptosis. Moreover, Stattic can also inhibit the DDR function of MV4-11 cells, delay the repair of DSBs, and thus enhance the induction of DSBs by VP-16 in AML cells. The mechanism may be related to the inhibition of the DDR pathway of AML cells. Our experimental results show that after treatment with Stattic, the level of ATM mRNA in leukemia cells is lower, and after Stattic and VP-16 treatment of cells, the expression of BRCA1, BARD1, RAD51, and polδ in the HR pathway are all downregulated. However, we also found that Stattic alone or in combination with VP-16 can upregulate the expression of some genes in the HR pathway, such as BRCA2 and RPA. This result seems to contradict the delay in repairing DSBs. We speculate that the dysregulation of various signaling molecules during HR repair is related to this paradox. In general, after DSBs occur in the cell, the damaged DNA ends are cut into 3′ single strands and combined with RPA to stabilize the single strand structure; next, RAD51 replaces RPA to form a RAD51-ssDNA complex and a D-loop at the DNA end. Finally, polδ performs DNA strand extension to complete DNA repair. It is noteworthy that assembly of the RAD51–ssDNA complex requires the participation of the following molecules: BRCA1, BRCA2, and RDA51 homologs (BARD1, RAD51D, RAD51B, and RAD51C) (Symington and Gautier, 2011; Iyama and Wilson, 2013; Kakarougkas and Jeggo, 2014; Fouquin et al., 2017). During this process, the increased expression levels of RPA and BRCA2 are conducive to the normal HR repair pathway. However, the downregulation of RAD51 and polδ expression downstream of RPA in the HR pathway may affect the normal repair of DSBs. Because the process of RAD51 replacing RPA requires the participation of BRCA1 and RAD51 homolog BARD1, the decrease in BRCA1 and BARD1 expression is not conducive to the successful assembly of RAD51–ssDNA complex and thus affects the normal progression of HR. However, the specific mechanism by which Stattic regulates the expression of genes such as BRCA1, BARD1, RAD51, and polδ mRNA still needs further study. In our experiments, we only showed changes in mRNA expressions of several HR-related factors; and our next step is to explore the effect of STAT3-inhibition on DDR pathway *in vivo*.

Besides hindering the repair of DSBs by affecting the HR pathway, Stattic can also induce DSBs. We speculate that Stattic affects the repair of spontaneously damaged DNA by accumulating DSBs in FLT3-ITD AML cells by inhibiting the HR repair pathway. This is because FLT3-ITD AML cells already have higher levels of DSBs. The continuous activation of the FLT3 receptor can activate the downstream PI3K/AKT, JAK/STAT, and Ras/MAPK pathways (Sellar and Losman, 2017). The RAS/PI3K/STAT pathway actively promotes the generation of ROS and then spontaneously induces the production of DSBs (Sallmyr et al., 2008).

Stattic can inhibit DDR function and hinder the repair of DSBs, thereby enhancing the sensitivity to chemotherapy drugs. Most traditional chemotherapeutic drugs kill leukemia cells by inducing DSBs, the accumulation of which leads to apoptosis (Goldstein and Kastan, 2015). Relapsed FLT3-ITD AML patients had an increased FLT3-ITD/FLT3-WT ratio in blasts of bone marrow as well as other new abnormalities in cellular and molecular level, which may be related to the HR pathway activity after DSBs AML cells. It may be related to LOH as well (Rebechi and Pratz, 2017). The results of this study provide a theoretical basis for the clinical treatment of FLT3-ITD AML by using STAT3 inhibitors combined with traditional chemotherapy drugs. This strategy could overcome the drug resistance of FLT3-ITD AML cells and improve the clinical prognosis of these patients.

In this study, we aims to explore the effect of Stattic on FLT3-ITD mutation AML cell lines (MV4-11) and the underlying mechanisms. We found Stattic can inhibit MV4-11 cell line proliferation and promote cell apoptosis, arrest cell cycle at G0/G1. Meanwhile, we also found the Stattic treatment suppress the HR pathway, which may be the mechanism of Stattic induced apoptosis. Our further investigation also found that Stattic can inhibit FLT3-WT cell line proliferation and promote cell apoptosis (Supplementary Figures S1, S2) by down regulate expression of STAT3-pi (Supplementary Figure S3); however, by comparing IC50 and apoptosis rate, we found that MV4-11 is more sensitive to Stattic than FLT3-WT cell line. Whether our conclusion from FLT3-ITD AML cells is applicable for multiple cell lines and animal models warrants further investigation.

## Data Availability Statement

The data presented in the study are deposited in the https://www.ncbi.nlm.nih.gov/sra/?term=PRJNA685978# repository, accession number PRJNA685978.

## Author Contributions

YXL and YL designed the study, conducted the experiments, and analyzed the data. BL, YSL, YY, and YX cultured cells. JZ supervised the study and wrote the manuscript. XZ corrected and approved the final version of the manuscript. All authors read and approved the final manuscript.

## Conflict of Interest

The authors declare that the research was conducted in the absence of any commercial or financial relationships that could be construed as a potential conflict of interest.

## Supplementary Material

Supplementary Figure 1 Stattic inhibits the growth of HL60 cells and KG1a cells. **(A)** HL60 cells were treated with 0, 1, 2.5, 5, and 10 μM Stattic for 24 h (*n* = 3, IR of HL60 cells treated with 1, 2.5, 5, and 10 μM Stattic for 24 h were 12.33 ± 3.97%, 24.96 ± 3.11%, 79.17 ± 1.22%, and 90.35 ± 2.27% IC50 = 3.65 μM). **(B)** KG1a cells were treated with 0, 1, 2.5, 5, and 10 μM Stattic for 24 h (*n* = 3, IR of KG1a cells treated with 1, 2.5, 5, and 10 μM Stattic for 24 h were 7.12 ± 3.35%, 18.58 ± 4.11%, 34.45 ± 2.40%, and 61.86 ± 2.40% IC50 = 6.22 μM).

Supplementary Figure 2 Stattic promotes apoptosis in KG1a and HL60 cells. KG1a cells and HL60 cells were treated with 1, 2.5, and 5 μM Stattic for 24 h. Apoptosis were quantified by flow cytometry. Data from three replica plates were plotted. Data are shown as mean ± *SD* **(A,B)**.

Supplementary Figure 3 Inhibition of STAT3-pi by Stattic in KG1a and HL60 cells. Protein expression of STAT3-pi in KG1a and HL60 cells treated with different concentrations of Stattic (1,2.5, and 5 μM) for 24 h, as detected by western blotting **(A,B)**.
