# Supplementary figures and images for: Blocking DNA Damage Repair May Be Involved in Stattic (STAT3 Inhibitor)-Induced FLT3-ITD AML Cell Apoptosis
Source: Front Cell Dev Biol. 2021 Mar 16;9:637064. doi: 10.3389/fcell.2021.637064 (PMC8007876; doi:10.3389/fcell.2021.637064)

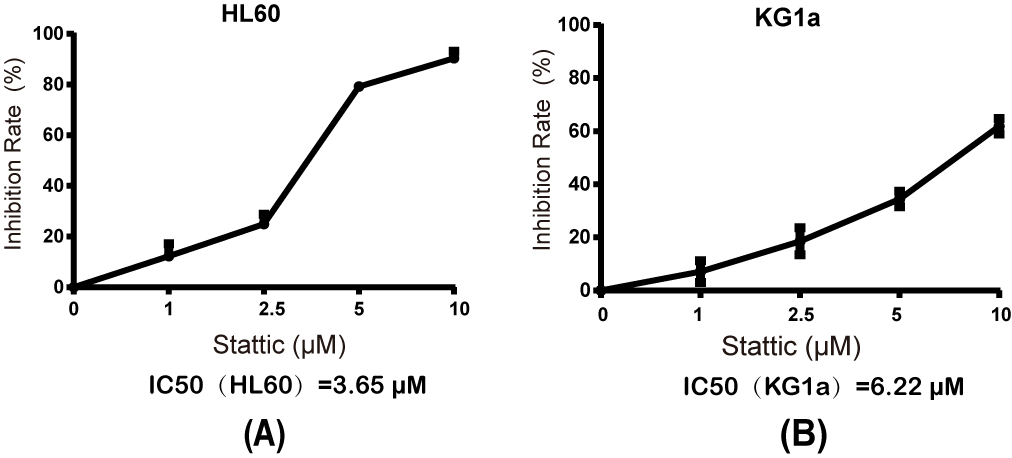

Supplement: Supplementary Figure 1 — Stattic inhibits the growth of HL60 cells and KG1a cells. (A) HL60 cells were treated with 0, 1, 2.5, 5, and 10 μM Stattic for 24 h (n = 3, IR of HL60 cells treated with 1, 2.5, 5, and 10 μM Stattic for 24 h were 12.33 ± 3.97%, 24.96 ± 3.11%, 79.17 ± 1.22%, and 90.35 ± 2.27% IC50 = 3.65 μM). (B) KG1a cells were treated with 0, 1, 2.5, 5, and 10 μM Stattic for 24 h (n = 3, IR of KG1a cells treated with 1, 2.5, 5, and 10 μM Stattic for 24 h were 7.12 ± 3.35%, 18.58 ± 4.11%, 34.45 ± 2.40%, and 61.86 ± 2.40% IC50 = 6.22 μM). [file Image_1.TIF]

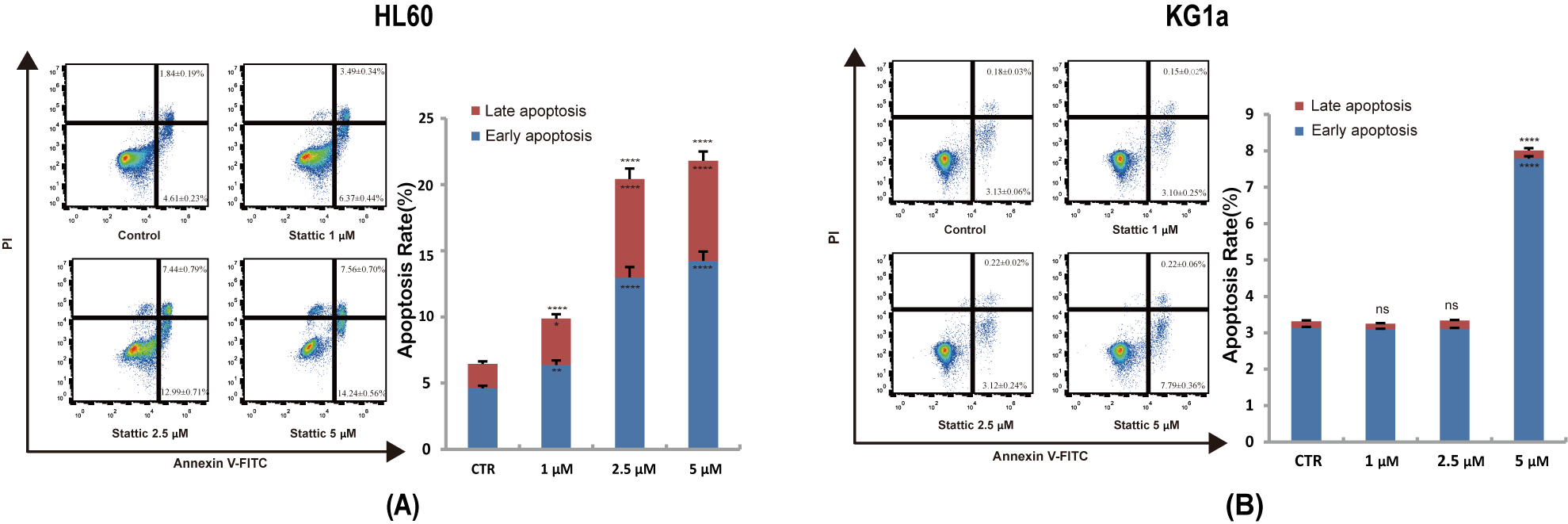

Supplement: Supplementary Figure 2 — Stattic promotes apoptosis in KG1a and HL60 cells. KG1a cells and HL60 cells were treated with 1, 2.5, and 5 μM Stattic for 24 h. Apoptosis were quantified by flow cytometry. Data from three replica plates were plotted. Data are shown as mean ± SD (A,B). [file Image_2.TIF]

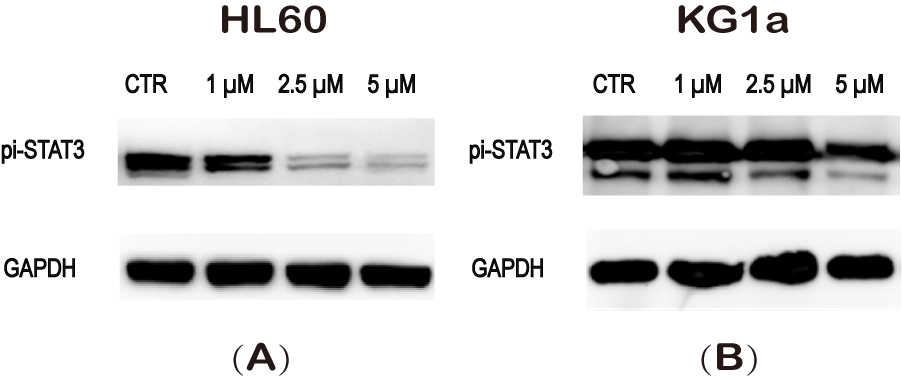

Supplement: Supplementary Figure 3 — Inhibition of STAT3-pi by Stattic in KG1a and HL60 cells. Protein expression of STAT3-pi in KG1a and HL60 cells treated with different concentrations of Stattic (1,2.5, and 5 μM) for 24 h, as detected by western blotting (A,B). [file Image_3.TIF]
